# Tracking Pathways Linking Obesity with Heart Failure

**DOI:** 10.3390/nu17071250

**Published:** 2025-04-03

**Authors:** Eleni Manta, Panagiotis Iliakis, Christos Fragoulis, Ioannis Leontsinis, Ioannis Stamoulopoulos, Christina Chrysohoou, Konstantinos Tsioufis

**Affiliations:** First Department of Cardiology, Hippokration General Hospital, Medical School, National and Kapodistrian University of Athens, 114 Vasilissis Sofias Avenue, 11527 Athens, Greece

**Keywords:** obesity, heart failure, inflammation, congestion, weight loss, GLP-1 receptor agonists, bariatric surgery

## Abstract

Obesity can cause the onset of heart failure and exacerbate the status of the pre-existing disease. Through intricate pathways, obesity activates hormonal factors that encourage the development of inflammation and lead to increased congestion. Consequently, this complex parallel pathophysiological cascade contributes to the echocardiographic and clinical signs of heart failure. In these patients, obesity frequently coexists with nutritional and muscular profile abnormalities that manifest as cachexia or sarcopenia. Patients with heart failure have a higher chance of surviving when obesity is treated. Interventional, pharmaceutical, and dietary strategies are used as forms of therapy. This review delves into the evaluation of the relationship between obesity and heart failure, and it targets to highlight the therapeutical impact of weight-loss programs on cardiac function in individuals with heart failure and obesity.

## 1. Introduction

Obesity is characterized by an excess accumulation of body fat that poses a health risk [1]. The Body Mass Index (BMI) is commonly used to assess body fat, considering an individual’s somatometric features. A BMI greater than 25 kg/m^2^ is considered overweight, while a BMI above 30 kg/m^2^ is classified as obese [2]. It appears that the distribution of adipose tissue is just as significant in obesity as its presence. Abdominal obesity cannot be adequately indicated by BMI alone. A straightforward technique for determining abdominal obesity is waist circumference, which has been highly correlated with cardiovascular and all-cause mortality [3]. Therefore, a waist circumference of 102 cm or more for men and 88 cm or more for women is considered abdominal obesity [3]. An additional indicator that illustrates the distribution of body fat is the ratio of hip to waist circumference (waist–hip ratio) [3]. The waist–hip ratio is calculated by dividing the waist circumference by the hip circumference. According to the World Health Organization (WHO), a waist–hip ratio of more than 0.90 for men and more than 0.85 for women is indicative of abdominal obesity [4].

The prevalence of obesity and being overweight are exponentially increasing, among adults, adolescences, and children. Between 1990 and 2022, the proportion of adults with obesity rose from 7% to 16%, while the percentage of children and adolescents with obesity grew from 2% to 8% globally [1]. Obesity is highly associated with the development and progress of other traditional cardiovascular risk factors, such as type II diabetes mellitus, hyperlipidemia, and hypertension. In addition, obesity is considered an independent risk factor for cardiovascular disease and death [5]. Notably, abdominal obesity seems to be a risk factor for cardiovascular disease that is independent of the BMI, as it has a substantial correlation with insulin resistance, metabolic disorders, and hypertension [6]. Therefore, the waist circumference and waist–hip ratio are useful for evaluating cardiovascular risk in addition to the BMI, as excess abdominal fat, especially visceral fat, raises the risk of heart disease by promoting atherosclerosis and inflammation [6].

Heart failure (HF) is a clinical condition caused by a structural or functional defect of the heart that causes high intracardiac pressures and insufficient cardiac output during exercise and/or rest [7]. It can be classified based on the left ventricular ejection fraction (LVEF) into three categories: HF with a reduced ejection fraction (HFrEF, where LVEF is ≤40%), HF with a mildly reduced ejection fraction (HFmrEF, with LVEF ranging from 41–49%), and HF with a preserved ejection fraction (HFpEF, where LVEF is ≥50%) [7]. HF is a serious public health concern that affects millions of people globally and causes a high rate of morbidity and mortality. HF affects 1–3% of the adult population, and its mortality rates remain high, with a 30-day mortality rate of approximately 2–3%, and a 5-year mortality rate ranging from 50–70% following hospitalization for HF [8].

This review aims to explore the relationship between obesity and HF by assessing the contribution of obesity to the onset and progression of HF. Furthermore, the potential benefits of non-pharmaceutical and pharmaceutical weight loss interventions in our arsenal will be described, in improving cardiac function and outcomes in patients with obesity and HF.

## 2. The Link Between Obesity and Heart Failure

**Figure 1:**
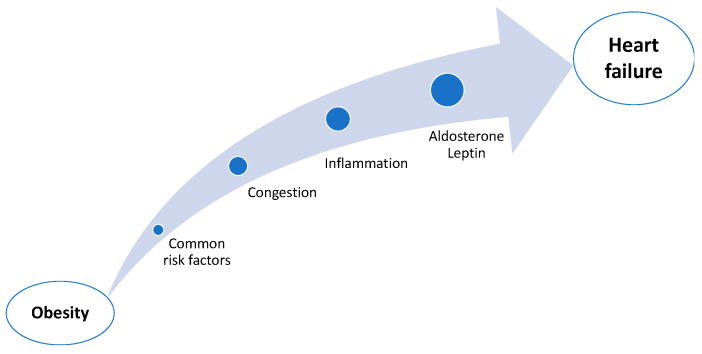
Pathophysiological mechanisms of obesity-related heart failure.

A great series of observational studies have documented obesity being a significant risk factor for the development of HF [9]. A greater BMI is linked to an increased risk of incident HF, and this is mostly seen in individuals with HFpEF as opposed to those with HFrEF [10]. Individuals who are overweight or obese account for up to 80% of patients with HFpEF in large multicenter studies [11]. Additionally, the meta-analysis conducted by Aune and colleagues demonstrated that for every 5 kg/m^2^ increase in the BMI, the risk of HF rises by 41% [12]. On the other hand, the HF incidence has been associated with abnormalities in healthy weight maintenance and physical exercise levels throughout adulthood [13]. The link between obesity and HF is reciprocal; obesity raises the risk of developing HF and also worsens the condition, once it has already occurred. This is derived from various pathophysiological alterations linked to obesity, which will be discussed in more detail below (Figure 1).

### 2.1. Common Risk Factors

Obesity and HF have always been linked to similar risk factors. These might include the co-occurrence of conditions including coronary artery disease, arterial hypertension, and diabetes mellitus [14]. Apart from the common risk factors, the risk of obesity-related HF is greatly influenced by environmental variables, such as unhealthy dietary patterns, physical inactivity, and social and economic status [15]. The impact of the genetic variables that may contribute to the development of obesity-induced HF is currently being studied. The genetic foundation of both general and abdominal obesity has been uncovered in part via genomewide association studies [16]. The contribution of these genetic factors in the susceptibility and progression of HF remains to be determined.

### 2.2. Congestion

An increased body mass due to obesity leads to an aberrant intravascular volume distribution because of a reduced venous capacity, as well as an increased blood and plasma volume, leading to increased filling pressures and increased left-ventricle end-diastolic pressure [17]. Furthermore, the left and right ventricles become dysfunctional due to pericardial constriction and increased stiffness brought on by epicardial fat [18]. Additionally, an increased BMI results in the mechanical constriction of the chest wall, which decreases left ventricular relaxation [14]. The aforementioned conditions lead to hemodynamic congestion and, consequently, HFpEF [18].

### 2.3. Inflammation

Adipose tissue, particularly located in the abdominal region, appears to be a significant factor in the development of obesity-related HF. Visceral obesity provides an ideal environment for the accumulation of chemokines and adipokines, resulting in the development of insulin resistance, leading to an increase in the number and activation of both inflammatory and immune system cells within the myocardium, resulting in inflammation and immunological dysfunction [19]. The heart muscle changes maladaptively in response to this dysfunction, resulting in hypertrophy, elevated myocardial cell tension, and eventually myocardial fibrosis [18].

### 2.4. Aldosterone, Leptin

Obesity is associated with increased levels of aldosterone and leptin. As leptin plays a neurotransmitting role, notifying the brain of the total fat amount stored in adipose tissue, it is associated with adverse effects on the brain areas linked to energy intake, which contributes to energy balance. These effects are generated by the binding of leptin to sympathetic neurons in the spinal cord and to melanocortin receptors in the hypothalamus [20]. Since people with obesity paradoxically have higher leptin levels, it has been suggested that leptin resistance develops, inhibiting the hormone’s ability to regulate hunger [21]. Several studies demonstrate that obesity also activates the renin–angiotensin–aldosterone pathway, resulting in increased aldosterone levels [21]. This activation happens mostly due to the renal-associated renin production, as a result of the interaction with the sympathetic nervous system. Another factor is the buildup of fat surrounding the kidney, which causes the kidney to compress and release renin (‘fatty kidney’) [21]. Furthermore, it appears that adipocytes contain an inherent renin–angiotensin–aldosterone pathway that generates angiotensinogen and angiotensin II and therefore aldosterone [21]. Leptin can also directly enhance the production of aldosterone by stimulating the adrenal glands [22]. The consequences of elevated levels of aldosterone and leptin are sodium and water retention, an increased intravascular volume, increased arterial stiffness, reduced left ventricular relaxation, and the eventual emergence of a HF clinical manifestation [14].

## 3. Frailty, Sarcopenia, and Cachexia

Frailty is described as a condition that increases a person’s vulnerability to stimuli [23]. It is estimated that up to 45% of individuals with HF are affected [24]. Frailty is very common in the elderly. Although there are few studies managing HF in older adults, therapy should be considered according to the guidelines [25]. It is linked to a lower tolerance for HF therapy as well as an elevated risk of hospitalization and mortality [26]. Sarcopenia is defined as the loss of skeletal muscle mass, quality, and function [27]. It is usually related to age, but chronic conditions such as HF can precipitate sarcopenia at a younger age [27]. Sarcopenia occurs in 20–50% of patients with HFrEF and is associated with increased morbidity and mortality [7]. Cachexia is a metabolic syndrome characterized by unintended edema-free weight reduction that primarily consists of muscle loss rather than fat loss, along with anorexia [28]. It is specifically defined as a reduction in dry body weight of at least 5% throughout the previous 12 months, affecting 5–15% of patients with HFrEF, and it is associated with worse survival outcomes [7].

Since relevant diagnostic scores have been mostly generated for patients with cancer, the criteria for detecting these two disorders in HF patients are restricted. The dry body weight loss previously indicated for cachexia, together with decreased handgrip strength and decreased walking speed for sarcopenia, are among the diagnostic criteria that have been recommended for individuals with HF [29]. Therefore, since sarcopenia and cachexia are linked to negative clinical outcomes, it is deemed reasonable to treat the aforementioned issues. Despite the lack of pertinent clinical research, it is advised that these patients’ diets be supplemented with protein to achieve a daily protein consumption of 0.8–1.1 g/kg [29], in conjunction with exercise that focuses on muscle strengthening [7,30].

## 4. The Obesity Paradox

Observational studies have shown that individuals who are overweight or obese may have a better prognosis regarding cardiovascular health, compared to individuals with a lower than normal weight [31]. The obesity paradox occurs in both HFrEF and HFpEF patients [32], with women seeming to be more affected than men [33].

The obesity paradox can be explained initially by the use of the BMI as a determinant for the diagnosis of obesity. The BMI is not a reliable indicator of the body composition with respect to the ratio of mass to fat. The type and location of adipose tissue play a pivotal role in the understanding of the obesity paradox, as subcutaneous, compared to abdominal, fat is associated with a lower cardiometabolic risk [34]. In contrast to the BMI, an increased waist–hip ratio, signaling the presence of central adipose tissue accumulation, appears to be more associated with a higher mortality [34].

Another possible explanation for this phenomenon is that obesity provides protection against malnutrition and the ensuing disorders of sarcopenia and cachexia, both of which have detrimental effects on cardiovascular outcomes [34]. As a result, patients with obesity exhibit greater tolerance to exercise, appearing to be a contributing factor to higher survival rates, compared to HF patients without obesity [34]. Additionally, the poor prognosis of individuals with HF is influenced by decreased insulin and glucose levels, which seem to be elevated in people with obesity [34]. Furthermore, regardless of body weight, individuals who exercise consistently seem to have greater survival rates, suggesting that regular physical activity is highly significant [30,35].

Body composition assessments may provide a better understanding of the obesity paradox. The analysis of body composition, such as dual-energy X-ray absorptiometry (DXA) and bioelectrical impedance analysis (BIA), helps differentiate between lean mass and adiposity, suggesting that the observed longevity advantages may be driven by more muscle mass rather than extra fat [36].

Nonetheless, it appears that several confounding variables raise doubts about the validity of the obesity paradox in HF patients. In contrast to observational data that suggested a protective effect, the employment of Mendelian randomization in several studies demonstrated that a higher genetically predicted BMI increases both the probability of developing HF and the mortality rates [37,38,39]. Similarly, the obesity paradox was challenged by Fall et al., who used Mendelian randomization to show that increased adiposity is directly connected to poorer HF outcomes [40]. Unintentional weight loss in HF may be another confounder of the obesity paradox. Unintended weight loss frequently represents cachexia, and therefore is a poor prognostic sign, especially in HF patients with obesity [41]. In contrast to regulated weight loss achieved via food and exercise, unintentional weight loss is caused by malabsorption and systemic inflammation, which raises the risk of death and functional impairment [42]. Accordingly, we can conclude that the retention of lean muscle mass may be a protective factor of obesity in HF, rather than just excess fat.

## 5. Therapeutic Interventions

As previously demonstrated, obesity is a major risk factor for HF, making its management essential for enhancing the cardiovascular health and quality of life in affected individuals. Managing obesity in HF patients is a complicated and multifaceted issue that necessitates a holistic strategy, combining lifestyle changes, medication, and occasionally surgical solutions (Table 1).

### 5.1. Lifestyle Modifications and Dietary Interventions

Adjusting lifestyle habits, especially through dietary changes, is fundamental in the therapeutical management of obesity. According to the American Heart Association, individuals with a BMI of 35 kg/m^2^ or higher should aim to lose at least 5% to 10% of their body weight, towards enhancing their cardiometabolic health and lowering the likelihood of cardiovascular outcomes [43]. Calorie restriction is a widely used method, generally suggesting a daily energy deficit of 500–750 kcal, which can result in a weight reduction of 1–2 pounds each week [44]. This method has been found to enhance the cardiac performance, especially in individuals with HFpEF. In the SECRET trial, a dietary approach involving calorie restriction led to a weight loss of 7 kg, improved 6 min walk test, increased peak oxygen uptake, and a reduction in the left ventricular mass among patients with HFpEF [45]. Nevertheless, the data supporting dietary changes in HFrEF are not strong, as many studies tend to be either small or not randomized, emphasizing the necessity for additional research in this field.

Certain dietary patterns may have an important role on the prevention and treatment of HF. The DASH (Dietary Approaches to Stop Hypertension) diet, that consists of foods low in sodium and high in potassium and fiber, lowers blood pressure, lessens fluid retention, and enhances heart performance in HF patients [46]. The Mediterranean diet, which is high in fruits, vegetables, whole grains, and healthy fats, is associated with reduced inflammation, enhanced endothelial function [47], and a decreased incidence of HF [48]. Additionally, plant-based diets may lessen oxidative stress and systemic inflammation [49], whereas high-protein diets may assist the maintenance of lean muscle mass in HF patients with sarcopenia. On the other hand, Western diets that are heavy in processed foods, refined sugars, and saturated fats have been connected to worsened HF outcomes by increasing obesity, insulin resistance, and myocardial stress [50]. One more parameter to evaluate is that HF patients with obesity frequently exhibit micronutrient deficiencies as a result of poor food quality, chronic inflammation, drug usage, and altered metabolism [51]. In conclusion, optimizing the HF treatment requires tailored nutritional plans that include nutritional supplements and take into account comorbidities and the necessary fluid balance.

There are several practical obstacles to implementing dietary recommendations for individuals with HF and obesity. Food preferences, long-standing dietary habits, and the psychological effects of dietary limitations can all make patient adherence challenging [52]. Moreover, it might be difficult for these patients to adhere to the suggested diets due to socioeconomic challenges, such as restricted availability to healthy foods and financial limitations [53]. Effective implementation is further hampered by shortcomings in the healthcare system, such as limited access to dietitians, nonpayment for nutrition advice, and time restraints during clinical appointments [54]. A multidisciplinary approach, patient education, and customized, practical meal programs that strike a balance between weight control and nutritional sufficiency are necessary to address these issues [53].

Exercise is essential for the management of obesity-related HF, as it enhances cardiac function, metabolic health, and cardiorespiratory fitness. By improving the left ventricular performance and stroke volume, exercise encourages favorable cardiac remodeling, which can be especially advantageous in HFpEF [14]. Additionally, sarcopenic obesity can be reversed through resistance exercise, which preserves the lean muscle mass [30]. Structured exercise programs, like cardiac rehabilitation, have been demonstrated to improve the quality of life, lower hospitalizations, and increase the long-term survival in HF patients with obesity [55].

### 5.2. Pharmacological Interventions

Recently, pharmacological therapies targeting obesity in patients with HF have garnered considerable interest, especially with the introduction of glucagon-like peptide-1 (GLP-1) receptor agonists. Liraglutide has been evaluated in various trials involving patients with HF. The FIGHT trial assessed liraglutide in individuals with advanced HFrEF, but the findings were not conclusive, showing not significant enhancements in clinical outcomes [56]. Conversely, the LIVE trial demonstrated that liraglutide led to a decreased body weight and better glycemic control in HF patients, although it did not result in significant improvements in left ventricular function [57].

Semaglutide, another GLP-1 receptor agonist, has demonstrated encouraging results in decreasing the body weight and enhancing the quality of life in individuals with obesity who are diagnosed with HFpEF. The STEP-HFpEF trial, a landmark trial in the field, involved 529 participants with a BMI of 30 kg/m^2^ or higher and demonstrated that a once-weekly subcutaneous injection of semaglutide resulted in an additional 11% reduction in body weight compared to a placebo, along with notable advancements in the Kansas City Cardiomyopathy Questionnaire Clinical Summary Score (KCCQ-CSS), 6 min walk test, and decreases in high-sensitivity C-reactive protein (hs-CRP) and N-terminal pro-B-type natriuretic peptide (NT-proBNP) levels [58]. The results indicate that GLP-1 receptor agonists may serve as an important choice in our quarry, namely obesity in patients with HFpEF, especially for those in which type 2 diabetes mellitus co-exists [59].

An alternative pharmacological treatment is tirzepatide, a dual agonist for glucose-dependent insulinotropic polypeptide (GIP) and GLP-1 receptors, demonstrating greater weight loss effectiveness compared to semaglutide in HF. Although there is a scarcity of evidence regarding its efficacy in patients with HF, its mechanism of action indicates that it could have favorable effects in enhancing metabolic profiles and lowering the cardiovascular risk factors [60]. Nonetheless, additional research is necessary to assess its efficacy and safety in individuals with HF.

Future epidemiological research is essential in order to evaluate GLP-1 receptor agonists’ long-term safety and efficacy in various HF subgroups. GLP-1 receptor agonists have been demonstrated to improve cardiovascular outcomes, glucose regulation, and weight reduction [61,62]. In addition, they seem to have a major effect on cardiac metabolism and structural remodeling over time in HF, as they improve endothelial function, lower insulin resistance, and increase myocardial glucose utilization—all of which may help reduce cardiac stress and inflammation [63]. However, their effects on hospitalization rates, cardiac function, and fluid balance require further study, especially in light of the possible natriuretic and diuretic side effects that may affect the volume status in HF patients [64]. In order to guide individualized treatment strategies and enhance the long-term outcomes, future longitudinal cohort studies and randomized controlled trials should examine the advantages, risks, and best use of GLP-1 receptor agonists for individual patients, especially considering the high prevalence of obesity and diabetes in HF populations.

Anti-inflammatory agents and metabolic modulators are becoming a new weapon in our quiver, due to their actions on pathways such as myocardial remodeling, chronic inflammation, and metabolic dysfunction. Anti-inflammatory treatments, such as colchicine and Interleukin-1 (IL-1) inhibitors (anakinra, canakinumab), attempt to restore endothelial function and lessen systemic inflammation, which may help HF patients with metabolic dysfunction [65]. Metabolic modulators, including the mitochondrial-targeted agent trimetazidine, may improve cardiac energy metabolism and lessen fibrosis and myocardial stress [66]. Even though these medications appear promising, further clinical research is required to determine their long-term safety, effectiveness, and best usage in various HF subgroups.

### 5.3. Bariatric Surgery

Bariatric surgery has been proven to be a successful and lasting approach for achieving substantial weight loss in suitable individuals with severe obesity (BMI ≥ 35 kg/m^2^). Moreover, bariatric surgery reduces cardiac workload, enhances diastolic function, and promotes favorable ventricular remodeling through long-term weight loss, the decreased accumulation of myocardial fat, and increased metabolic efficiency [67]. Especially in HFpEF populations with obesity, longitudinal studies show that post-operative patients had better overall cardiac performance, lower filling pressures, and less left ventricular mass [67]. A major retrospective analysis that included 298,101 HF patients with a BMI ≥ 35 kg/m^2^ demonstrated that those who received bariatric surgery experienced significantly improved health outcomes compared to those who did not. In particular, this type of surgery has been linked to a reduction in the overall mortality, fewer hospitalizations related to HF, and a decreased occurrence of atrial fibrillation [68].

Even though the results are promising, there is a deficiency of randomized controlled trials assessing the effects of bariatric surgery specifically in patients with HF. The ongoing BRAVE trial is designed to fill this void by comparing bariatric surgery with medical weight management in patients with high-risk cardiovascular conditions, including symptomatic HF patients (clinicaltrials.gov, NCT04226664) [69]. The findings from this trial may offer clearer evidence regarding the effectiveness of bariatric surgery in treating obesity among HF patients.

It is worth mentioning that the eligibility and results of bariatric surgery for HF patients with obesity are significantly influenced by socioeconomic factors, according to the epidemiological data. Racial and ethnic minorities, those with lower incomes, and those with poor access to healthcare are less likely to be referred for bariatric surgery even when they match the eligibility requirements [70]. Post-operative results are also influenced by socioeconomic variables, since patients from underprivileged origins may have more complications, follow dietary recommendations less closely, and have less access to multidisciplinary care [71]. Future studies should concentrate on the long-term cardiovascular benefits, efforts to enhance outcomes across a variety of HF groups, and fairness in surgical access.

### 5.4. Challenges and Considerations

One of the significant difficulties in handling obesity in patients with HF is the coexistence of cachexia, which can occur alongside obesity. This contradictory situation makes the management of these patients challenging, since conventional weight loss methods may worsen cachexia. Thus, a refined, well-rounded approach is of great importance, ensuring a balance between the necessity for weight reduction and the avoidance of muscle loss. Possible interventions for cachexia include appetite enhancers, exercise programs, anabolic substances, and dietary supplements, though none have been shown to be reliably effective [72].

Another important aspect to consider is how cardiac steatosis and lipotoxicity contribute to obesity-related cardiomyopathy. The accumulation of excessive fat in cardiomyocytes may result in myocardial dysfunction, regardless of the overall metabolic disturbances. This underscores the necessity of focusing not just on the total body weight but also on the distribution and influence of fat within the heart. Magnetic resonance imaging and spectroscopy have been proven useful methods for evaluating cardiac steatosis and informing the treatment approaches [73].

## 6. Limitations

The presence of predominantly observational studies about the relationship between obesity and HF is one of the limitations of the results drawn from the present literature review. Further crucial information that might assist the safer generalizability of the findings would be obtained by conducting relevant randomized trials or subgroup analyses according to gender, age, and comorbidities.

## 7. Conclusions

Obesity is a crucial cardiometabolic risk factor for both HFrEF and HFpEF, highlighting that its management is essential to improve the cardiovascular health and quality of life in affected individuals. Obesity and HF are linked by pathophysiological pathways that involve humoral and mechanical processes. A thorough comprehension will aid in the more efficient and timely treatment of obesity-related HF. Achieving weight loss and enhancing cardiovascular health involves lifestyle changes, medication options, and bariatric surgery, all of which are vital components, as the goal of this holistic approach and management of the affected individuals is the loss of fat and not muscle tissue. GLP-1 receptor agonists like semaglutide and liraglutide have demonstrated effectiveness in helping individuals with obesity and HFpEF improve their weight and quality of life; therefore, they should become an integral part of our therapeutic approach for these patients. Bariatric surgery provides a long-term solution for substantial weight reduction in qualified individuals, though some patients face difficulties in accessing this procedure.

Future studies should aim to fill the knowledge gaps concerning the interplay between obesity, nutrition, and HF outcomes. Ensuring that all HF patients have fair access to efficient weight loss options, including bariatric surgery, should be prioritized to allow everyone to benefit from these advancements. By combining these strategies, we can enhance the treatment of obesity in HF patients, ultimately improving their quality of life and long-term health outcomes.

## Figures and Tables

**Table 1:** The main trials of therapeutic interventions for the treatment of obesity-related heart failure.

Intervention	HFrEF	HFpEF
Diet		**SECRET** ○ calorie restriction ○ N = 100 patients ○ −7 kg ○ improvement of symptoms, LVM
GLP-1 receptor agonists	**FIGHT (liraglutide)** ○ N = 300 patients ○ −1.9 kg ○ not conclusive	**STEP-HFpEF (semaglutide)** ○ N = 529 patients ○ −11% of body weight ○ improvement of symptoms, hs-CRP, NT-proBNP
	**LIVE (liraglutide)** ○ N = 241 patients ○ −2.2 kg ○ no improvement of LVEF	
Bariatric surgery	**BRAVE** N = 2000 patients ongoing	

N = number, LVEF = left ventricular ejection fraction, LVM = left ventricular mass, hs-CRP = high-sensitivity C-reactive protein, NT-proBNP = N-terminal pro-B-type natriuretic peptide.

## Abbreviations

The following abbreviations are used in this manuscript:

BMI	Body Mass Index
WHO	World Health Organization
HF	Heart failure
LVEF	Left ventricular ejection fraction
HFrEF	Heart failure with reduced ejection fraction
HFmrEF	Heart failure with mildly reduced ejection fraction
HFpEF	Heart failure with preserved ejection fraction
DXA	Dual-energy X-ray Absorptiometry
BIA	Bioelectrical Impedance Analysis
DASH diet	Dietary Approaches to Stop Hypertension diet
GLP-1 receptor	Glucagon-like peptide-1 receptor
KCCQ-CSS	Kansas City Cardiomyopathy Questionnaire Clinical Summary Score
hs-CRP	High-sensitivity C-reactive protein
NT-proBNP	N-terminal pro-B-type natriuretic peptide
GIP receptor	glucose-dependent insulinotropic polypeptide receptor
IL-1	Interleukin-1
